# Reliability and validity of self-report questions for assessing levels of physical activity and sedentary time in adult childhood cancer survivors

**DOI:** 10.1186/s13102-024-00851-3

**Published:** 2024-03-06

**Authors:** Laura Jess, Marianne Jarfelt, Maria Bäck

**Affiliations:** 1 Department of Oncology, Institute of Clinical Sciences, Sahlgrenska Academy, University of Gothenburg, Sahlgrenska University Hospital, Blå stråket 2, 413 45 Gothenburg, Sweden; 2 Närhälsan Bollebygd Rehabilitation Clinic, Bollebygd, Sweden; 3 The Long-Term Follow-Up Clinic for Adult Childhood Cancer Survivors and Cancer Rehabilitation, Sahlgrenska University Hospital, Gothenburg, Sweden; 4 Department of Occupational Therapy and Physiotherapy, Sahlgrenska University Hospital, Gothenburg, Sweden; 5 Department of Health, Medicine and Caring Sciences, Division of Prevention, Rehabilitation and Community Medicine, Unit of Physiotherapy, Linköping University, Linköping, Sweden; 6 Department of Molecular and Clinical Medicine, Institute of Medicine, Sahlgrenska Academy, University of Gothenburg, Gothenburg, Sweden

**Keywords:** Accelerometer, ActiGraph, Measurement tool, Paediatric cancer survivorship, Surveys

## Abstract

**Background:**

Regular physical activity and limited sedentary time are recommended for adult childhood cancer survivors. The Swedish National Board of Health and Welfare designed a questionnaire to assess levels of physical activity (BHW-Q), including two questions: one on vigorous physical activity (BHW-Q VPA) and one on moderate physical activity (BHW-Q MPA). Furthermore, a single-item question was developed to measure sedentary time (SED-GIH-Q). These questions are recommended for clinical practice and have been found valid for the general population but have so far not been tested in adult childhood cancer survivors. The aim of the study was to assess test–retest reliability, agreement and criterion-related validity of the BHW-Q and the SED-GIH-Q in adult childhood cancer survivors.

**Method:**

A non-experimental methodological study. In total 60 participants (50% women), median age 28 (min-max 18–54) years were included at the Long-Term Follow-Up Clinic at Sahlgrenska University Hospital. Participants were instructed to wear an accelerometer for seven days, and to answer the BHW-Q and the SED-GIH-Q before and after the seven days. Test-retest reliability and criterion-related validity comparing the BHW-Q and SED GIH-Q with accelerometer data were calculated with weighted Kappa (k) (agreement) and by using Spearman´s rho (r) (correlation).

**Results:**

Test-retest reliability regarding the SED-GIH-Q showed a high agreement (k = 0.88) and very strong correlation (*r* = 0.93), while the BHW-Q showed a moderate agreement and moderately strong correlation, BHW-Q VPA (k = 0.50, *r* = 0.64), BHW-Q MPA (k = 0.47, *r* = 0.58). Both the agreement and the correlation of the criterion-related validity were interpreted as fair for the BHW-Q VPA (k = 0.29, *r* = 0.45), while the agreement for BHW-Q MPA was interpreted as low (k = 0.07), but the correlation as fair (*r* = 0.37). The agreement of the SED-GIH-Q (k = 0.13) was interpreted as low and the correlation as poor (*r* = 0.26).

**Conclusion:**

These simple questions assessing physical activity and sedentary time can be used as screening tools in clinical practice to identify adult childhood cancer survivors in need of support to increase physical activity level. Further development is needed on the design of a sufficiently valid question measuring sedentary time.

**Trial registration:**

This research project was registered in the Swedish National Database of Research and Development; identifier 275251, November 25, 2020. https://www.researchweb.org/is/vgr/project/275251.

## Background

In Sweden the overall survival rates for childhood cancer survivors have improved to over 80% in the past decades [1]. Seven out of 10 childhood cancer survivors suffer from complications of the disease and cancer treatment over time [2]. This includes a significant risk of metabolic syndrome [3], cardiovascular disease [4], secondary cancer [5, 6] and neurocognitive impairments [7]. Regular physical activity is associated with a lower risk of morbidity and mortality in several chronic diseases, including cancer survivors [8, 9]. Moreover, sedentary time has been identified as a serious health concern [10]. To achieve the positive health benefits of physical activity, childhood cancer survivors are recommended to follow the physical activity guidelines of the general population [11], including moderate-intensity cardiorespiratory physical activity (MPA) for at least 150 min a week or 75 min of vigorous-intensity physical activity (VPA), or an equivalent combination of MPA and VPA throughout the week. In addition, the amount of sedentary time should be limited and replaced by activity of any intensity [12]. The guidelines for physical activity were updated in 2020 and the recommended 10-minute bouts were removed due to new evidence supporting that any length of physical activity contributes to health effects [13]. Healthcare professionals have an important role in supporting adult childhood cancer survivors to be physically active and to reduce sedentary time [14]. An adequate assessment of physical activity is needed to identify individuals in need of support to meet physical activity recommendations and for determining trends in physical activity over time or evaluation of effects of physical activity interventions [15]. Self-report questionnaires are common and cost-effective measurements of physical activity under free-living conditions but have a risk of recall bias and over- and underestimation. Alternatively, objective measurement with accelerometry is reliable and valid and provides detailed information on intensity, frequency and duration, but for healthcare practice the administration may be too complex and costly [15, 16].

To the best of our knowledge, there is no available valid and reliable questionnaire that can evaluate physical activity levels and sedentary time in adult childhood cancer survivors. The Swedish National Board of Health and Welfare (BHW) designed a questionnaire with two indicator questions on physical activity (BHW-Q) [17]. Furthermore, The Swedish School of Sport and Health Science (GIH) has designed a question for the assessment of sedentary time (SED-GIH-Q) [18]. These questions are recommended as screening tools in healthcare, and they have been found valid for use in a clinical setting to identify people that are insufficiently physically active [17, 18]. However, results from studies aimed at evaluating the validity of physical activity questionnaires in one population cannot be directly extrapolated to other populations or other geographical regions [19]. Furthermore, it is important to assess test-retest reliability to determine whether a questionnaire is consistent over time [20]. Therefore, the aim of this study was to assess test–retest reliability, agreement and criterion-related validity of the BHW-Q about physical activity and the SED-GIH-Q about sedentary time for adult childhood cancer survivors.

## Methods

Methodological non-experimental study.

### Participants

Adult childhood cancer survivors were recruited from the Long-Term Follow-Up Clinic (LTFU) at the Department of Oncology, Sahlgrenska University Hospital in Gothenburg, Sweden, between November 2021 and September 2022. Inclusion criteria were age ≥ 18 years, diagnosed with cancer at < 18 years of age. Exclusion criteria were recurrence or secondary malignancy at study inclusion, individuals with physical or cognitive impairments or insufficient knowledge of the Swedish language.

### Procedure

A physician screened eligible patients for participation during a physical or digital visit at the LTFU and provided written study material and information that the study coordinator would call within a week. Participants who signed informed consent received the study questionnaire, wear-time diary and accelerometer by post. The questionnaire covered background data (e.g. age, gender, level of education, employment status, cancer diagnosis), and contained the BHW-Q and the SED-GIH-Q. The questionnaire also included a question about whether participants had received any information about physical activity recommendations during or after treatment. The participants were asked to fill in the questionnaire and to wear the accelerometer on their right hip during waking hours for seven days. They were instructed to take off the accelerometer during water-based activities (e.g. showering). Participants were asked to report the accelerometer wear time in a diary. After seven days, the participants were asked to fill in the BHW-Q and the SED-GIH-Q again. After completion, the accelerometer and questionnaires were sent back to the LTFU at the Department of Oncology, Sahlgrenska in stamped addressed envelopes.

### Measurements

#### Physical activity and sedentary time questionnaire

The physical activity questionnaire (BHW-Q) contains two questions [17].

*BHW-Q VPA: During a regular week, how much time do you spend exercising at a level that makes you short of breath, for example running, fitness class, or ball games?*

The questions are answered by pre-defined categories 1–6:

1 = 0 min, 2 = less than 30 min, 3 = 31–60 min, 4 = 61–90 min, 5 = 91–120 min, 6 = more than 120 min.

*BHW-Q MPA: During a regular week, how much time are you physically active in ways that are not exercise, for example walks, bicycling, or gardening? Add together all activities lasting at least 10 min.*

The questions are answered by pre-defined categories 1–7:

1 = 0 min, 2 = less than 30 min, 3 = 31–60 min, 4 = 61–90 min, 5 = 91–150 min, 6 = 151–300 min, 7 = more than 300 min.

We decided to add a modified question of the BHW-Q MPA including all physical activity BHW-MPAtot, due to the removal of the recommended 10-minute bouts in the updated guidelines in 2020 [13].

*BHW-Q MPAtot: During a regular week, how much time are you physically active in ways that are not exercise, for example walks, bicycling, or gardening?*

The questions are answered by the same pre-defined categories as *BHW-Q MPA 1–7.*

GIH’s question for assessing sedentary time (SED-GIH-Q) [18].

How much time do you spend sitting during a normal day not counting sleep?

The question is answered by pre-defined categories 1–7:

1 = Basically all day,2 = 13–15 h, 3 = 10–12 h, 4 = 7–9 h, 5 = 4–6 h, 6 = 1–3 h, 7 = never.

#### Accelerometer

Physical activity was measured objectively with the ActiGraph GT3X-BT accelerometer (ActiGraph, Pensacola, FL, USA) with normal filter settings. The accelerometer was used as a criterion-related validity instrument. The software ActiLife (version 6.13.4) was used to initialise, extract, and analyse the raw data from the accelerometer. The GT3X-BT measures electrical impulses from accelerations with a triaxial capacitive microelectromechanical system (MEMS) sensor. Data were collected with a sampling rate of 30 Hz and the raw data were converted to 60 s epochs in the unit counts per minute (cpm) for the vector magnitude that combined cpm from three axes into one outcome.

The Choi algorithm was used for calculating non-wear time [21], defined as consecutive zero counts for at least 90 min, while allowing a short time interval with nonzero counts lasting up to 2 min. Furthermore, non-wear time was compared with the time recorded in the diaries. A minimum of 600 min and at least four valid days, was required for being included in the analysis [22]. The Sasaki accelerometer cut-points for vector magnitude were used to differentiate between intensities of physical activity [23]. A cutoff of < 149 cpm was used to define sedentary time; light-intensity physical activity (LPA) 150–2689 cpm, MPA 2690–6166 cpm, VPA ≥ 6167 cpm. The weekly median time in wear time, LPA, MPA, VPA and sedentary time was calculated by dividing the sum of each variable by valid days and multiplying it by 7. For the purpose of comparing questionnaire data with accelerometer data, the pre-defined categorical answers from the questionnaire (BHW-Q VPA 1–6; BHW-Q MPA 1–7) were transformed to categorical minute scores by taking the mean of the interval for each category, e.g. 45 min for the category 31–60 min. The continuous accelerometer data were categorised into the same categories as the BHW-Q and GIH-SED-Q answer options. The “Activity minutes” were calculated by multiplying accelerometer data VPA by two, plus MPA, to generate an outcome of the total physical activity volume.

### Statistical analysis

Statistical analyses were performed using SPSS, version 25.0 (IBM Corp. Armonk, NY, USA). Descriptive statistics was used to calculate demographics of the population and physical activity levels and sedentary time assessed with the questionnaires and accelerometer. After testing all variables for normal distribution with the Shapiro-Wilk test, the data were treated as non-parametric. Ordinal data were described with median and Q1-Q3, nominal data in numbers and percentages while interval data were described in median and min-max. To analyse test-retest reliability and criterion-related validity between the accelerometer and questionnaire Spearman’s rho (r) and linearly weighted Kappa coefficient (k) was used. To interpret weighted Kappa and Spearman’s rho the following criteria were used: A Kappa coefficient of 1 means perfect reliability, > 0.81 is considered almost perfect reliability, 0.61–0.8 as substantial, 0.41–0.6 as moderate, 0.21–0.4 as fair and < 0.2 as weak reliability [24]. Spearman’s rho correlation coefficient can assume values between − 1 and 1, where 1 indicates a perfect correlation and − 1 indicates a perfect negative correlation. The closer to 0, the weaker the correlation. At least 0.8 indicates very strong correlation, 0.6–0.8 as moderately strong, 0.3–0.5 as fair and < 0.3 indicates a poor correlation [25].

## Results

### Participants

**Fig. 1:**
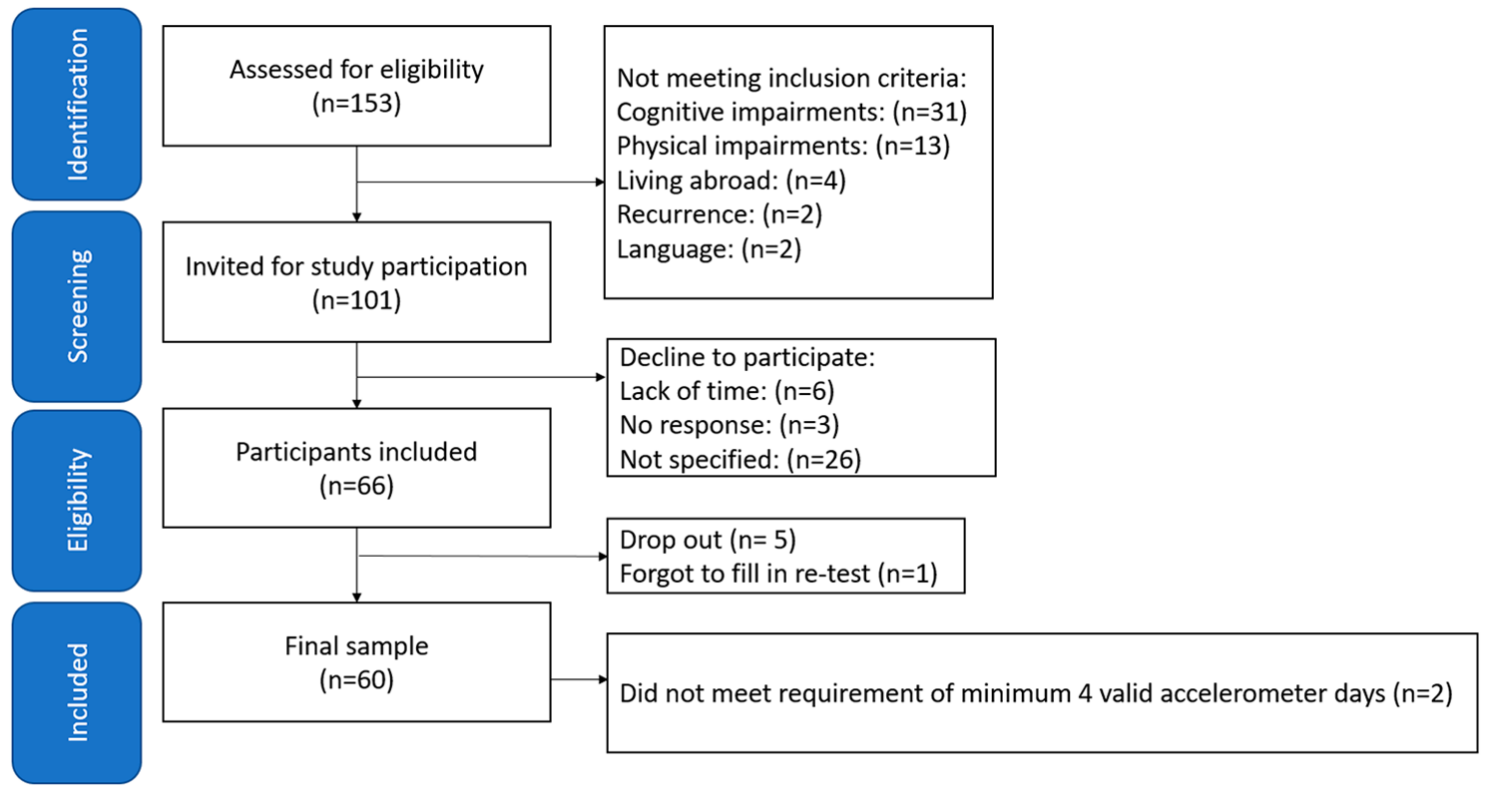
Flowchart of the recruitment procedure

A total of 153 adult childhood cancer survivors were screened for eligibility. Forty-eight individuals were excluded due to the exclusion criteria and an additional four individuals were living abroad at study and were excluded for practical reasons. In total, 101 individuals were invited and 66 agreed to participate in the study. Reasons for declining were lack of time (*n* = 6), no response (*n* = 3) or not specified (*n* = 26), flowchart of the recruitment procedure is shown in Fig. 1. Five participants dropped out after inclusion and one participant forgot to fill in the test-retest questionnaire. A total of 60 patients (50% women), with a median age of 28 (min-max 18–54) years, were included in the analysis. Median age at diagnosis was 6 (min-max 0–17) years. For characteristics of the study population, see Table 1.

**Table 1:** Demographic and clinical characteristics of the study population (*n* = 60)

**Adult childhood cancer survivors**	
Gender, women, n (%)	30 (50)
Age at diagnosis, years, median (min-max)	6 (0–17)
Age at study inclusion, years, median (min-max)	28 (18–54)
Years since cancer treatment, median (min-max)	19 (2–49)
**Highest level of education n (%)**	
Not completed upper secondary school	2 (3.3)
Completed upper secondary school or Higher Vocational Education	31 (51.7)
University degree	27 (45)
**Work situtation n (%)**	
Employed	42 (70)
Studying	12 (20)
Long-term sick leave	4 (6.6)
Other	2 (3.3)
**Home situation n (%)**	
Married/co-habitation	29 (48.3)
Living alone	29 (48.4)
Living with parents	2 (3.3)
**Living n (%)**	
Rural area	6 (10)
Small town	33 (55)
Larger city	21 (35)
**Diagnosis n (%)**	
Leukemia (ALL, AML, CML)	28 (46.7)
Lymphoma (HL, NHL)	11 (18.3)
Sarcoma (bone or soft tissue)	10 (16.7)
CNS tumor (malignant brain tumor, spinal tumor)	6 (10)
Other (Neuroblastoma, Juvenile angiofibroma, Retinoblastoma, Wilms tumor)	5 (8.3)
**Treatment n (%)**	
Chemotherapy	57 (95)
Surgery	21 (35)
Radiation	32 (53.3)
Have received advice about Physical activity n (%)*	
No	36 (60)

Data presented as median (min-max) or as number *n* and percent (%)

ALL– acute lymphoblastic leukaemia

AML - acute myeloid leukaemia

CML - Chronic myelogenous leukaemia

HL - Hodgkin’s lymphoma

NHL - non-Hodgkin’s lymphoma

*According to what the participant reported in the questionnaire

### Physical activity questionnaires

Participants reported that they spent a median of 45 (Q1-Q3 0–75) min/w in VPA and a median of 165 (Q1-Q3 45–225) min/w in MPA at follow-up. Participants reported a median of 8 (Q1-Q3 5–11) hours of sedentary time a day, see Table 2.

**Table 2:** Descriptive data from the questionnaires at baseline and follow-up (*n* = 60)

	Baseline	Follow-up
Question		
BHW-Q VPA (min/week)	45 (15,105)	45 (0,75)
BHW-Q MPA (min/week)	165 (45,225)	165 (45,225)
BHW-Q MPAtot (min/week)	165 (45,225)	165 (75,225)
SED-GIH-Q (hours/day)	8 (5, 11)	8 (5, 11)
Activtity minutes* (min/ week)	255 (105, 375)	225 (135, 360)
Activity minutes** (min/ week)	255 (135, 375)	255 (165, 375)

Data presented as median (Q1-Q3); BHW-Q VPA: The Swedish National Board of Health and Welfare question about exercise. BHW-Q MPA: The Swedish National Board of Health and Welfare question about everyday physical activity (10-minute bouts). BHW-Q MPAtot: The Swedish National Board of Health and Welfare question about everyday physical activity (all activity). SED-GIH-Q: GIH’s question for assessing sedentary time. * BHW-Q VPA x2 + BHW-Q MPA. ** BHW-Q VPAx 2 + BHW-Q MPA (total)

### Accelerometer data

Participants spent a median of 7 (Q1-Q3 0–40) min/w in VPA and a median of 292 (Q1-Q3 181–484) min/w in MPA and a median of 8 (Q1-Q3 6–9) hours of sedentary time a day, see Table 3.

**Table 3:** Physical activity assessed with accelerometer (*n* = 58)

Variable	Median (Q1-Q3)	Mean (SD)
Total wear time (min/day)	852 (812, 894)	855 (67)
VM counts (counts/minute)	342 (291,435)	368 (126)
Steps (steps/day)	7039 (5350, 9688)	7788 (3220)
LPA (min/week)	2461 (2005, 2873)	2455 (607)
MPA (min/week)	292 (181, 484)	339 (202)
VPA (min/week)	7 (0, 40)	26 (34)
MVPA bouts (min/week)	123 (38, 215)	155 (135)
Activity minutes* (min/week)	152 (65,271)	190 (166)
Activity minutes** (min/week)	327 (208,327)	386 (228)
Sedentary (hours/day)	8 (6, 9)	8 (2)

Data presented as median (Q1-Q3) and mean (standard deviation); VM counts: Vector magnitude counts; LPA: Light physical activity; MPA: Moderate physical activity; VPA: Vigorous physical activity. MVPA bouts: moderate to vigorous physical activity added for every 10 min. * BHW-Q VPA x2 + BHW-Q MPA. ** BHW-Q VPAx 2 + BHW-Q MPAtot.

### Test-retest reliability

Test-retest reliability showed a moderate agreement for the BHW-Q VPA (k = 0.50, *p* < 0.001), the BHW-Q MPA (k = 0.47, *p* < 0.001) and the BHW-Q MPAtot (k = 0.54, *p* < 0.001). The SED-GIH-Q showed a high agreement (k = 0.88, *p* < 0.001). The correlation for the SED-GIH-Q was very strong (*r* = 0.93, *p* < 0.001), while the correlation for the BHW-Q was moderately strong (*r* = 0.64, *r* = 0.58 and *r* = 0.65, *p* < 0.001), for more details see Table 4.

**Table 4:** Test-retest self-reported physical activity and sedentary time (*n* = 60)

Question	Kappa (95% CI)	p (k)	Spearman’s rho	p (rho)
BHW-Q VPA	0.50 (0.35–0.64)	< 0.001	0.64**	< 0.001
BHW-Q MPA	0.47 (0.30–0.63)	< 0.001	0.58**	< 0.001
BHW-Q MPAtot	0.54 (0.39–0.69)	< 0.001	0.65**	< 0.001
SED-GIH-Q	0.88 (0.80–0.96)	< 0.001	0.93**	< 0.001

BHW-Q VPA: The Swedish National Board of Health and Welfare question about exercise. BHW-Q MPA: The Swedish National Board of Health and Welfare question about everyday physical activity (10-minute bouts). BHW-Q MPAtot: The Swedish National Board of Health and Welfare question about everyday physical activity (all activity). SED-GIH-Q: GIH’s question for assessing sedentary time

### Criterion-related validity accelerometry

The criterion-related validity, comparing self-reported data with accelerometer data, was interpreted as fair for the BHW-Q VPA (k = 0.29, *p* < 0.001), while the criterion-related validity was interpreted as low for the BHW-Q MPA (k = 0.07, *p* = 0.132), the BHW-Q MPAtot (k = 0.09, *p* = 0.103) and the SED-GIH (k = 0.13, *p* = 0.074). The correlations for BHW-Q VPA, BHW-Q MPA, BHW-Q MPAtot were fair (*r* = 0.45, *p* < 0.001, *r* = 0.37, *p* = 0.005, *r* = 0.35, *p* = 0.006), while the correlation of SED-GIH-Q was poor (*r* = 0.26, *p* = 0.05), see Table 5.

**Table 5:** The correlation and agreement between the questionnaires and categorical accelerometer data (*n* = 58)

Question	Kappa (95% CI)	p (k)	Spearman’s rho	p (rho)
BHW-Q VPA	0.29 (0.13–0.44)	< 0.001	0.45**	< 0.001
BHW-Q MPA	0.07 (-0.02-0.16)	0.132	0.37**	0.005
BHW-Q MPAtot	0.09 (0.00- 0.17)	0.103	0.35**	0.006
SED-GIH-Q	0.13 (-0.02-0.28)	0.074	0.26**	0.05

BHW-Q VPA: The Swedish National Board of Health and Welfare question about exercise. BHW-Q MPA: The Swedish National Board of Health and Welfare question about everyday physical activity (10-minute bouts). BHW-Q MPAtot: The Swedish National Board of Health and Welfare question about everyday physical activity (all activity). SED-GIH-Q: GIH’s question for assessing sedentary time

### Meeting physical activity guidelines

According to the questionnaire 45% (10-minute bouts) and 48% (total) of the participants did meet the physical activity guidelines, compared to 60% (total) and 31% (10-minute bouts) using the accelerometer data. A total of 43% spent at least 8 h a day in sedentary time according to the accelerometer data, compared to 58% according to the questionnaire. At baseline, 40% of the participants reported that they had received information on physical activity recommendations, Table 1.

## Discussion

To the best of our knowledge, reliable and valid questionnaires assessing physical activity level and sedentary time in adult childhood cancer survivors are lacking. Our study will contribute new knowledge to the field by demonstrating an almost perfect test-retest reliability of the SED-GIH-Q and a moderate test-retest reliability of the BHW-Q. The criterion-related validity was poor to fair, which is similar to previous studies in other populations.

The study demonstrated a high agreement (k = 0.88) and a very strong correlation (*r* = 0.93) for the test-retest reliability of the SED-GIH-Q, which is slightly higher compared to a previous study in elderly people from the general population showing a substantial agreement (k = 0.77) and a very strong correlation (*r* = 0.86) [26]. The SED-GIH-Q could be valuable for screening purposes in clinical practice as the single-item question can easily be integrated. However, the criterion-related validity of the SED-GIH-Q was interpreted as low and the correlation was shown as poor, which may partly be explained by the formulation of the question that refers specifically to sitting time and as such does not capture the whole and complex construct of sedentary time [27]. A recent review suggested asking about the duration of seated activities instead of total sitting time, as people rarely reflect on their posture but instead label their activities based on higher-order goals (e.g. time spent driving or processing information) [27]. Another possible explanation for the low criterion-related validation could be that the responses were initially divided into pre-determined categories and further categorised during the analyses, where for instance 1–3 h became 2 h. This might have introduced bias in terms of capturing the exact time spent sedentary. However, our results of the agreement of the SED-GIH-Q (k = 0.13, *r* = 0,12) are similar to a validation study performed in individuals who survived colon cancer, which compared data from an ActiGraph GT3X + accelerometer and the Marshall Domain-Specific Sitting Questionnaire and found that both the correlation and the agreement between the two methods were poor (*r* = 0.19, ICC = 0.16) [28]. As the ActiGraph GT3X can’t distinguish between different postures the activPAL might have been a better choice [29]. Nevertheless, our research findings also align with a current study in a middle-aged Swedish population, which investigated the criterion-related validity of the SED-GIH-Q by using the activPAL3 micro as the criterion measurement and found a moderate correlation (*r* = 0.31) and a poor agreement (k = 0.12) [26]. This highlights that measuring sedentary time with a questionnaire is challenging and needs further development. Also, further investigations are needed to evaluate if the SED-GIH-Q is able to detect changes over time.

All three questions included in the BHW-Q showed a moderate agreement and a moderately strong correlation. However, test-retest reliability was slightly better in the updated question, where the 10-minute bouts were removed. This may indicate that remembering the total amount of physical activity tends to be more manageable than recalling specific bouts. Our results were similar to a study in survivors of adult cancer, which found a fair to excellent reliability for the Activity Questionnaire for Adults and Adolescents (AQuAA) (ICC = 0.57 to 0.78) and a good to excellent reliability for the Physical Activity Scale for the Elderly (PASE) (ICC = 0.67 to 0.90) [30]. Similar to the other studies, we chose a period of 7 days between the repeated measurements in order to avoid recall bias but also to avoid clinical changes.

The agreement and correlation of the criterion-related validity was interpreted as fair for the BHW-Q VPA (*r* = 0.45), while the BHW-Q MPA and the BHW-Q MPAtot showed similar results with a low agreement and fair correlation (*r* = 0.36) respectively (*r* = 0.35), which is slightly better than in the original validation study of the BHW-Q (*r* = 0.31), which included 365 Swedish adults with a mean age of 51 years [17]. In a study of Boyle et al. [28] involving colon cancer survivors, accelerometer-based data were compared to the Godin Leisure-Time Exercise Questionnaire and found fair correlation (*r* = 0.51) [28], which indicates that exercise is a more consistent behaviour and may be easier to recall than everyday physical activity. The strengths of physical activity questionnaires are that they are feasible, cost-effective, and therefore easy to apply in clinical settings and in large sample studies. However, there may be a risk of recall bias, risk of over- and underestimating physical activity, social desirability and differential bias [31]. These questions might be used to provide a rough indication in clinical practice to identify individuals in need of support to increase physical activity level, however accelerometer assessment is recommended for a detailed assessment of physical activity and sedentary time.

According to the accelerometer data 40% of the participants did not meet the physical activity guidelines, compared to 27.5% of adults in the general population [32]. This finding confirms previous research which indicated that adult childhood cancer survivors tend to have lower levels of physical activity compared to the general population [33, 34]. A systematic review looking into the validity and reliability of different physical activity measurements found that self-reported physical activity estimates in general are higher than when directly measured (e.g., accelerometer, doubly-labelled water) [35]. In this study it was found that 60% of the participants met the physical activity guidelines according to the accelerometer while only 48% of the participants met the physical activity guidelines according to the questionnaire. This suggest that the participants in this study have underestimated their physical activity levels, which is a surprising finding as individuals typically overestimate their physical activity level [36]. This finding needs further investigation through additional research. Perception bias due to social comparison or personal expectations may be a possible explanation. In a recent qualitative study conducted by our research team [37], adult childhood cancer survivors could express feeling unable to match the physical activity levels of others. Comparing themselves to more active peers may lead them to perceive their own activity level as lower. Another explanation for the challenges in estimating physical activity levels is that participants may have had difficulties in correctly classifying and quantifying their physical activity levels. Only 40% of participants reported receiving physical activity guidelines, indicating a potential knowledge gap in differentiating between everyday physical activity, exercise, and sedentary time.

### Strengths and limitations

A strength of this study was using the accelerometry as reference method which is considered as the gold standard for measuring physical activity under free-living conditions. Nevertheless, activities such as swimming, biking and strength training can’t be assessed by the accelerometer which may result in differences in self-reported data compared to the data from the accelerometer [38]. Another strength was that we included adult childhood cancer survivors with various diagnoses, treatments, ages, and genders, which increases the generalisability to adult childhood cancer survivors in the clinical setting. However, this also results in a large symptom heterogeneity and increasing the number of subjects might have minimised the standard error of the mean [39]. Also, we excluded many individuals with cognitive issues, as the study was conducted during the pandemic and our ability to give on-site support was hampered. There is a need to evaluate this subgroup as well. Another limitation could be that this was a single centre study and physical activity behaviour might differ within Sweden and compared to other countries. Also, there are no validated accelerometer cut-points for adult childhood cancer survivors. It is important to be aware that the choice of accelerometer cut-points affects to what extent the participants reach the physical activity guidelines. We chose Sasaki’s accelerometer cut-points [23] as they are commonly used and therefore enable comparison with other studies. However, it would be beneficial to develop accelerometer cut-points specifically tailored to adult childhood cancer survivors in the future.

### Future perspectives

There is a need for further research about sedentary behaviour, as no questions to detect sedentary time appear to be sufficiently valid and meet the required standards. As only a few studies in survivors of adult cancer have assessed validity and reliability of physical activity questionnaires, most studies in cancer survivors and adult childhood cancer survivors tend to use questionnaires that are validated in other populations. Therefore, further research about how to optimally assess physical activity and sedentary time in adult childhood cancer survivors is needed.

## Conclusion

The SED-GIH-Q has shown a poor validity but an almost perfect reliability. The validity of the BHW-Q was low to fair, while the reliability was moderate. This simple questionnaire assessing physical activity and sedentary time can be helpful in clinical practice to identify adult childhood cancer survivors in need of support to increase physical activity level. However, accelerometer assessment is recommended for more detailed assessment of physical activity and sedentary time.

## Data Availability

Data from the current study is not publicly available due to ethical conditions but is available from the corresponding author on reasonable request.

## Abbreviations

LTFU: Long-Term Follow-Up Clinic
LPA: Light-intensity physical activity
MPA: Moderate-intensity physical activity
MVPA: Moderate and vigorous physical activity
VPA: Vigorous-intensity physical activity
